# The impact of oral rehabilitation with implants in nutrition and quality of life: A questionnaire-based survey on self-perception

**DOI:** 10.4317/jced.55647

**Published:** 2019-05-01

**Authors:** Érica Bugone, Cristina-Balensiefer Vicenzi, Moisés-Zacarias Cardoso, Luana Berra, João-Paulo de Carli, Ademir Franco, Luiz-Renato Paranhos, Maria-Salete-Sandini Linden

**Affiliations:** 1 Department of Prosthodontics, School of Dentistry, University of Passo Fundo, Brazil; 2 Department of Therapeutic Stomatology, Institute of Dentistry, Sechenov University, Russia; 3 Department of Preventive and Community Dentistry, Federal University of Uberlandia, Brazil

## Abstract

**Background:**

The association between tooth loss and masticatory problems may influence on food choices and consequently impact nutrition and quality of life. This study aimed to evaluate impact of oral rehabilitation with implants in nutrition and quality of life.

**Material and Methods:**

A prospective study was performed. The Questionnaire for Healthy Habits (QHH) and the Oral Health Impact Profile-14 (OHIP-14) tools were used to assess nutrition and oral health status, respectively. Oral implants were placed and the adjacent the bone was radiographically assessed. The mean outcomes of the QHH between pre- and post-rehabilitation periods were assessed with the Wilcoxon Signed Rank test. The OHIP-14 was assessed using Fisher’s exact test.

**Results:**

The implant surfaces showed a significant bone loss after six and 24 months of rehabilitation (*p*<0.001). There was no significant change in the masticatory pattern of patients (*p*>0.05). Nevertheless, the patients perceived a significant reduction in discomfort (*p*<0.02) when eating, after 24 months of the rehabilitation.

**Conclusions:**

These findings confirm the hypothesis that oral rehabilitation with implants may not trigger direct improvement in nutrition. However, it plays an especial role improving quality of life.

** Key words:**Dentistry, nutrition, oral implants, quality of life.

## Introduction

Population longevity is increasing worldwide and the mutilating dental practices of the last decades have affected a large number of elderly edentulous people. Along with this problem emerges the need for improving oral rehabilitation treatments to reestablish the masticatory condition of the population (1). Tooth loss is one of the main health problems due to its high prevalence (2) and direct impact in quality of life. Its impact may be expressed by the reduction of masticatory performance, phonetic impairments, nutritional deficiencies, decreased self-esteem and limited social integration (3).

It is known that tooth loss is preventable in most cases and occurs as a reflection of the accumulation of oral diseases throughout life, as a result of trauma, or even as an expression of cultural aspects. Mutilating practices, including unnecessary extractions – which are still common, are a mark of social inequality in several societies, in which poor populations suffer greater losses (2). The possibility of eating foods with different textures and nutritional values is the main benefit provided by natural teeth (4). Digestion begins in the oral cavity and its satisfactory progress depends on the presence of teeth and/or prosthesis. In this context, nutritional deficiencies may manifest as a consequence of tooth loss. More specifically, part of the food is eliminated without being digested and absorbed in the intestines.

Moreover, masticatory deficiencies force the individual to select foods that are easier to chew, resulting in an unbalanced diet that does not meet daily nutritional requirements (5). A diet with predominance of carbohydrates and less consistent foods may cause anemic and apathetic states, as well as masticatory muscle atrophy, which affects facial aesthetics and self-esteem. Good masticatory performance is the most difficult function to restore. Prior to the development of dental implants, the therapeutic options consisted in complete or partial removable prostheses and bridges (6). Recently, implant therapy became an alternative treatment (7) that may promote more stability, masticatory efficiency, safety (8), phonetic and aesthetic improvements and comfort (9,10).

The treatment with implant-supported prostheses has shown excellent prognoses (11). However, the maintenance of peri-implant bone is essential for long-term success (12). Several factors may cause peri-implant bone loss, such as distance between implants, previous or current periodontal disease, occlusal overload, peri-implant soft tissue quality, ratio of crown/implant length and microbiological control (8). Success behind the oral rehabilitation with implants is assessed from an equation that involves the implant itself, the peri-implant soft tissue, the prosthesis, and the self-perception of patients. These criteria are in constant debate, but the achievement and maintenance of osseointegration are acknowledged as crucial factors; therefore, marginal bone loss is an important factor (12). Based on the exposed, this study aimed to evaluate impact of oral rehabilitation with implants in nutrition and quality of life of patients via a questionnaire-based survey on self-perception.

## Material and Methods

-Ethical issues and study design

The present study was institutionally approved by the local committee of ethics in human research (protocol number: 047/2012) and it was carried out in full compliance with the Declaration of the World Medical Association of Helsinki. It is an observational and analytical study with prospective longitudinal design. Accordingly, the data was sequentially presented according to the Strengthening the Reporting of Observational Studies in Epidemiology (STROBE) statement (13).

-Participants

The sample consisted of patients older than 18 years ongoing oral rehabilitation with multiple or single implants at the institutional dental clinic. Lack of systemic diseases and continuous medication figured as incusion criteria. Patients that were not willing to participate as volunteers were excluded from the study. All the included patients signed an informed consent agreeing on participating in the study.

-Intervention and follow-up

The recruited patients underwent general dental procedures to make the oral environment adequate for receiving implants. After the surgical procedure for placing the implants, the osseointegration period was respected and the prostheses were placed on the implants. After rehabilitations, the patients were followed-up and evaluated clinically and radiographically for 24 months. The date of prostheses placement was set as baseline.

Periapical radiographs were standardly taken using the parallelism technique. After captured, the radiographic images were digitized and the peri-implant bone height of the patients was observed at each time (baseline, 6 months, 12 months, 18 months and 24 months). In order to standardize the observations, the implant platform was selected as fixed reference in relation to the adjacent bone.

-Implant-related variables

The reference was used to quantify and compare the marginal bone level on the mesial and distal surfaces of each implant with the marginal bone at the level of the implant platform. During this procedure, Image Tool™ (UTHSCSA, San Antonio, TX, USA) software was used. The measurements were obtained in pixels and converted to millimeters. In order to prevent the influence of radiographic distortion over the measurements, the previously known length of the implants was considered as reference. The criterion used to make inferences on the success of implant survival was the maximum cervical bone loss of 1.5 mm/year in the first year and 0.2 mm/year in the second year (14).

-Nutrition and quality of life-related variables

Concomitant with bone maintenance control, a nutritional analysis was performed to verify, through a validated questionnaire, the diet habits of the patients before and after rehabilitation. The Questionnaire for Healthy Habits (QHH) of the Brazilian Ministry of Health was used, which responses corroborate the recommendations for healthy eating. In parallel, the Oral Health Impact Profile-14 (OHIP-14) (15) questionnaire was used to investigate the real impact of oral conditions on the quality of life of patients rehabilitated with oral implants.

-Statistical analysis

The bone loss values at each stage and the results of the QHH were calculated using the Wilcoxon Signed Rank test, while the results of the OHIP-14 questionnaire were calculated and evaluated using Fisher’s exact test. The statistical software used was the Sigma Plot (Systat Software Inc., San Jose, CA, USA). Significance level was set at 5%, while the confidence level was set at 95%.

## Results

 Table 1 shows the description of the sample according to type of prosthesis and the location of implants installed. Overall, 58 implants were installed, from which 46 (79.3%) consisted of either a single element or components of fixed prostheses with two or three elements, located uniformly in the maxilla (31-53.4%) and the mandible (27-46.6%). For the single element prosthesis, 68% were installed in the maxilla.

**Table 1:**
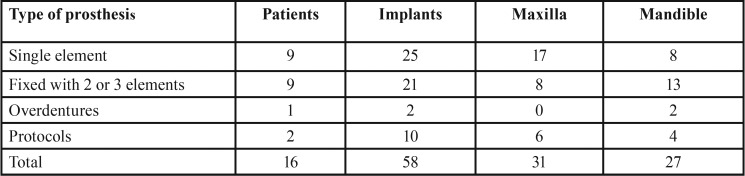
Descriptive analysis of the sample according to the type of prosthesis and location of implants.

The assessment of the 58 implants after six months of installation ( Table 2) revealed significant bone loss was observed on both the mesial and distal surfaces (*p*<0.001). After 24 months of follow-up, only 10 (17.2%) implants were reevaluated and significant bone loss was observed again on both surfaces ( Table 3) (*p*<0.001). The same accounted for the bone loss combining both surfaces ( Table 4) (*p*<0.001).

**Table 2:**
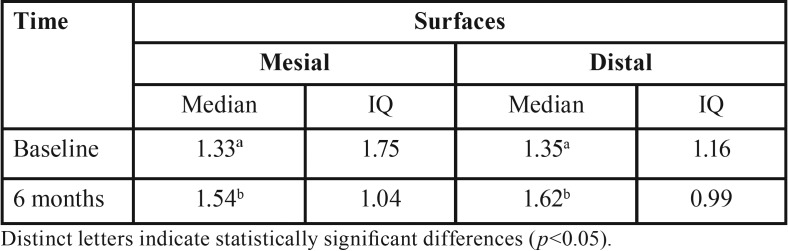
Median and interquartile (IQ) values of insertion depth (mm) of implants, after installation and at six months of follow-up, according to the surface (mesial and distal, n=58).

**Table 3:**
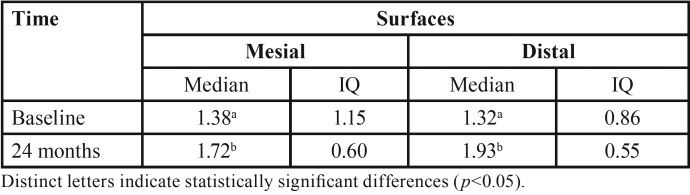
Median and interquartile (IQ) values of insertion depth (mm) of implants, after installation and at 24 months of follow-up, according to the surface (mesial and distal, n=10).

**Table 4:**
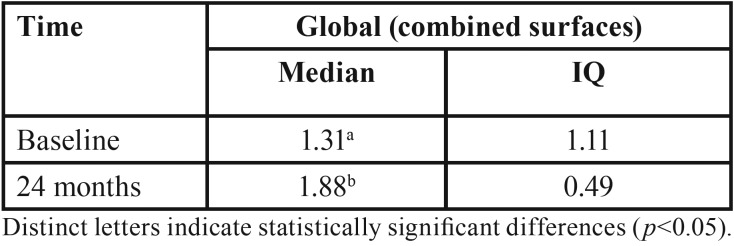
Median and interquartile (IQ) values of insertion depth (mm) of implants, after installation and at 24 months of follow-up (n=10).

According to the outcomes of the QHH tool, statisticaly significant differences were not observed when the masticatory pattern was compared between baseline and 24 months from baseline (*p*>0.05).

According to the OHIP-14 tool, the patients replied whether or not the absence of teeth compromised their performance in daily functions. A significant reduction of discomfort was perceived at 24 months in comparison to baseline (*p*<0.02).

## Discussion

Implant-supported prostheses figure amongst the most popular oral rehabilitation options nowadays, as it reestablishes masticatory, phonetic, and aesthetic functions, and allows patients to have a life similar to that before tooth loss. However, the greatest success of this rehabilitation option is reestablishing stomatognathic functions and peri-implant maintenance. The present study showed significant, but controlled, peri-implant bone loss. In 24 months, there was no change in the masticatory pattern of the patients, but there was a significant improvement in the quality of life of the individuals rehabilitated.

The main objective of oral rehabilitation is to improve the oral health and quality of life of the patient, considering that edentulism has a negative influence that implies psychosocial impairment and functional incompetence (16). Two recent longitudinal studies that used the Oral Health Impact Profile (OHIP) questionnaire observed a positive impact of oral rehabilitation on the quality of life of patients, as well as the reduction of functional limitations, physical pain, and psychological and social discomfort before and after treatment (16). The present study showed significant improvement in some areas, but in the overall score, the difference after rehabilitation was not significant.

Some studies have compared the impact of implant rehabilitation on the quality of life of patients through questionnaires and by including aspects such as functional limitation when eating, which was also evaluated in the present study. Such studies showed positive effects of this therapy, with an increase in the quality of life of patients and improvement of self-perception on daily activities (17,18). The results found corroborate those of this study, which showed a significant reduction in eating difficulties after 24 months of implant therapy.

Berretin-Felix *et al.* (19) (2017) evaluated the nutritional status of elderly patients whose removable prostheses were replaced with implants. The authors observed that the placement of prostheses on implants in the elderly did not modify their nutritional condition in short term, suggesting that the subjects had maintained the previous diet habits. In the same follow-up, a systematic review and meta-analysis observed improvement in nutrient intake and markers of nutritional status versus treatment with mandibular overdenture when compared to conventional total prosthesis. As a result, there was no significant difference in body mass index change between overdenture and conventional prosthesis wearers six months after treatment, and there was no significant difference in the change in albumin or vitamin B12 between the two treatments. Thus, the authors found that the modifying effect of this treatment on nutritional status may be limited and that a greater number of studies are required to evaluate its efficacy (20). Through the questionnaires and patient self-perception, the present study reported a reduction in eating difficulties, whereas after rehabilitation, the patients were able to eat foods they could not previously. However, none of the questionnaires could capture masticatory pattern change within 24 months, as in the studies aforementioned.

Two studies comparing the masticatory performance associated with different rehabilitation strategies in patients with edentulous mandibles observed that osseointegrated implants greatly improved the masticatory performance of all groups rehabilitated with implants when compared to the group of conventional complete dentures (21). A bibliographical review evaluated the improvement in chewing, bite force, patient satisfaction, and nutritional status in patients rehabilitated with upper or lower overdentures and observed high patient satisfaction regarding comfort, although not always accompanied by an improvement in general quality of life. For the authors, the treatment of implant-supported total prosthesis wearers improves masticatory efficiency and satisfaction and increases maximum bite force, but the improvement in the quality of life is uncertain (22).

The findings of the aforementioned studies differed from the present study, in which patients who returned for the 24-month follow-up did not show changes in their masticatory pattern, and this difference may be explained by two factors. First, most of the patients rehabilitated lost a few elements in the posterior region, unilaterally, considering 79.3% of either single element prostheses or components of fixed prostheses with two or three elements and only 20.7% of overdentures and protocols; thus, the changes in masticatory pattern went unnoticed by the patients. In a second moment, the evaluation method used by the present study resorted only to patient self-perception, subjectively. As the rehabilitations were smaller, such change might have been measured objectively and captured more easily. The variety of methods used to measure changes in masticatory performance, patient satisfaction, and nutritional status makes it difficult to compare the different results of the studies, so it would be pertinent to propose a standardized methodology to evaluate this issue (22).

The marginal bone around the crestal region of the implant is generally a significant indicator of peri-implant health. The method used to evaluate bone loss after healing is the standardized radiographic evaluation (with all the parameters of image acquisition controlled, including X-ray source and receiver), which was the same used in the present study (23,24). The literature presents different values to determine the success of implant survival and bone maintenance over the years. The criterion used in this study for maximum cervical bone loss was the one suggested by Albrektsson *et al.* (14) (1986) of 1.5 mm/year in the first year and greater than 0.2 mm/year in the following years. The same is a consensus for Misch et al. (23) (2008) regarding the health quality scale of the International Congress of Oral Implantologists – Pisa Consensus Conference. Such scale determines that the implant is successful and healthy when it presents less than 2.0 mm of radiographic bone loss compared to the surgical insertion of the implant. Silva *et al.* (11) (2011) also state that peri-implant bone loss is acceptable when it ranges from 1.5 to 2.0 mm in the first year of prosthetic restoration.

Trullenque-Eriksson and Guisado-Moya (25) (2014) evaluated the survival and marginal bone loss of 342 implants for 13 years using the radiographic method. The mean bone loss was 0.77±1.10 mm and it was greater than 3 mm in 2.5% of the implants analyzed. The potential factors for implant survival and marginal bone loss were smoking, osteopenia or osteoporosis, frequency of reassessment, implant surface, length, and position, and type of prosthesis. Pellicer-Chover *et al.* (26) (2014) evaluated and compared peri-implant health and marginal bone loss in 144 implants. As a result, they observed that at prosthetic loading, the probing depth was greater in immediate implants than late implants, with statistically significant differences. However, after 6 and 12 months, the differences between groups had disappeared. Bone loss was 0.54±0.39 mm for the immediate implants and 0.66±0.25 mm for the late implants.

The mean results found in the present study after six months of follow-up were 0.21 mm of loss in the mesial surface and 0.27 mm in the distal surface of the 58 implants. After 24 months of follow-up, 10 implants were reevaluated and the loss increased to 0.34 mm in the mesial surface and 0.61 mm in the distal surface, and the overall loss was 0.57 mm. These results are within the acceptable mean limits of bone loss for implant success, as observed in previous studies (14,23). In view of the variability found between the studies presented and our study, a standardization of examinations and measures is required to prevent discrepancies regarding the determination of success and the maintenance of bone height, considering that a misclassification may compromise the entire rehabilitation treatment. Finally, even if mean values or groups of implants are used, it is known that each implant should be monitored as an independent unit when assessing bone loss, otherwise the successful clinical evaluation loses credibility and there is a risk of an overestimated survival or neglect of failures, as observed by Misch *et al.* (23) (2008).

This study presents some limitations that should be considered, whereas the reduced number of patients with implants who returned for follow-up limited the inference of results, in addition to the limitations inherent to the longitudinal study design (e.g., cost, time, and absence of follow-up). However, we may also highlight the positive aspects of this research, which worked with the preparation of the oral environment prior to implant installation and the careful evaluation and analysis of osseointegration with a half-year follow-up of the elements rehabilitated. Other strengths of the study were the longer follow-up period than most studies, the use of validated instruments, and a radiographic protocol to evaluate bone loss, which included an imaging software. Finally, it is worth noting that our findings may contribute to reinforce those already mentioned in this discussion.

Tooth loss is still considered a public health problem due to its high prevalence, and aesthetic, functional, psychological, and social compromises. Edentulism may be reversed through dental rehabilitation treatments, and the prosthesis on implants is the most current and efficient of them, showing positive results in increasing masticatory effectiveness, safety, and improvement of psychological factors and patient self-esteem. The patients rehabilitated in this study did not identify significant changes in masticatory pattern, which may be related to rehabilitations with partial dentures and few elements. However, positive results may be observed in the significant reduction of discomfort and loss when eating, which are important indicators for improving the quality of life and nutritional status of the patient.

Although there was bone loss around the implants within the acceptable mean limits for implant success, the patients evaluated in this study showed improvement in quality of life after the prosthetic rehabilitation, including specific improvements for eating.
